# The Nature and Stability of Transition Metal‐Anchored Nitrogen‐Doped Graphene Single‐Atom Catalysts

**DOI:** 10.1002/chem.202501654

**Published:** 2025-07-14

**Authors:** Angelina N. van Dam, Pascal Vermeeren

**Affiliations:** ^1^ Department of Theoretical Chemistry Amsterdam Institute of Molecular and Life Sciences (AIMMS) Vrije Universiteit Amsterdam De Boelelaan 1108 Amsterdam 1081 HZ The Netherlands

**Keywords:** bonding mechanism, demetallation, density functional theory, Energy Decomposition Analysis, Single‐Atom Catalysis

## Abstract

Transition metal‐anchored nitrogen‐doped graphene single‐atom catalysts (SACs) represent an emerging class of catalysts that combine the advantages of both homogeneous and heterogeneous catalysis. To prevent demetallation and ensure catalyst stability, sufficiently strong bonds between the transition metal and the support are essential. We have quantum chemically analyzed the trend in bonding interaction between period 4 transition metals (TM = Ti, V, Cr, Mn, Fe, Co, Ni, Cu, and Zn) and the four‐nitrogen‐doped graphene support. We find that the metal–support interactions strengthen from Ti to Ni but weaken from Ni to Zn. Activation strain and Kohn‐Sham molecular orbital (KS‐MO) analyses reveal that this trend stems from changes in the interaction between the metal's 3*d*_xy_ atomic orbital and the nitrogen lone pair orbitals of the support. As we move along period 4, the bonding mechanism changes from an increasingly more stabilizing HOMO–LUMO interaction (TM = Ti‐Ni), due to the higher effective nuclear charge of the metal, to a less favorable HOMO–SOMO (TM = Cu) and unfavorable HOMO–HOMO (TM = Zn) interaction, as a result of the filling of the metal's 3*d*_xy_ atomic orbital. This results in the observed strengthening, followed by weakening, of the metal–support interaction. These insights could guide the rational design of future single‐atom catalysts.

## Graphical abstract

Our quantum chemical analyses of the bonding trends between period 4 transition metals and nitrogen‐doped graphene support reveal that the metal–support interaction strengthens from Ti to Ni, then weakens from Ni to Zn. This trend arises from changes in bonding interaction between the transition metals’ 3*d*_xy_ orbital and nitrogen lone pairs of the graphene support.

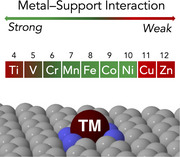

## 1 Introduction

Single‐atom catalysts (SACs) are an emerging class of catalysts that combine the advantages of homogeneous and heterogeneous catalysis,^[^ ^1^ ^]^ offering high catalytic activity, selectivity, stability, tunability, and efficient metal utilization.^[^ ^1a,b^ ^]^ SACs are characterized by an isolated catalytically active transition metal (TM) atom anchored in a solid support. Graphene is an excellent choice as a support material due to its stability, high conductivity, and large surface area.^[^ ^2^ ^]^ Additionally, the structural‐electronic properties of the graphene support can be fine‐tuned by doping with heteroatoms, particularly nitrogen, to alter the catalytic efficiency.^[^ ^3^ ^]^ In nitrogen‐doped graphene supports, the catalytically active TM atom is anchored in the nitrogen‐rich vacancies of the support, being stabilized by four TM─N bonds. These structural‐electronic features make SACs the surface analogues of organometallic complexes.^[^ ^1^, ^4^ ^]^ In recent years, SACs have been employed in various electrochemical and thermal reactions, such as the hydrogen evolution reaction,^[^ ^5^ ^]^ oxygen reduction reaction,^[^ ^6^ ^]^ cross‐coupling reactions,^[^ ^7^ ^]^ and cycloaddition reactions.^[^ ^8^ ^]^

The widespread use of SACs, however, remains limited due to the possible demetallation of the catalytically active TM atom during catalysis, which diminishes catalytic efficiency.^[^ ^9^ ^]^ Strong metal–support interactions are, therefore, crucial to prevent demetallation and maintain catalyst stability.^[^ ^1^, ^10^ ^]^ By constructing a thermodynamic cycle and using Pourbaix diagrams, Pacchioni et al.^[^ ^11^ ^]^ evaluated the stability of SACs in electrochemical reactions. They found that a key factor in determining the overall stability of a SAC was the binding strength of the catalytically active TM atom to the support. Hensen et al. and Senftle et al. used a combined density functional theory (DFT) and machine learning approach to screen the diffusion barrier of TMs on solid supports.^[^ ^12^ ^]^ Their findings further corroborate those from Pacchioni et al.,^[^ ^11^ ^]^ showing that the binding energy of the TM to the support is an important parameter for the height of the diffusion barrier. Thus, the likelihood of demetallation directly depends on the TM–support binding strength, and understanding these interactions is, therefore, essential for designing robust SACs.

In earlier studies, Di Valentin and co‐workers employed qualitative organometallic chemistry concepts, such as crystal and ligand field theory, along with a projected density of states analysis, to explore the bonding between various period 4 TMs and the nitrogen‐doped graphene support.^[^ ^13^ ^]^ Their findings suggested that the 3*d*_xy_ atomic orbital of the TM engages in a σ‐bonding interaction with the nitrogen lone pair orbital of the nitrogen‐doped graphene support. The 3*d*_xz_ and 3*d*_yz_ atomic orbitals of the TM, on the other hand, are predicted to interact with the π‐system of the support, while the 3*d*_z_^2^ and 3*d*_x_^2^_–y_^2^ remain nonbonding. Despite identifying various bonding interactions, quantitative insight into the importance of each bonding interaction on the stability of the SAC, and trends therein, remains unclear, which is crucial for the rational design of new SACs.

**Scheme 1:**
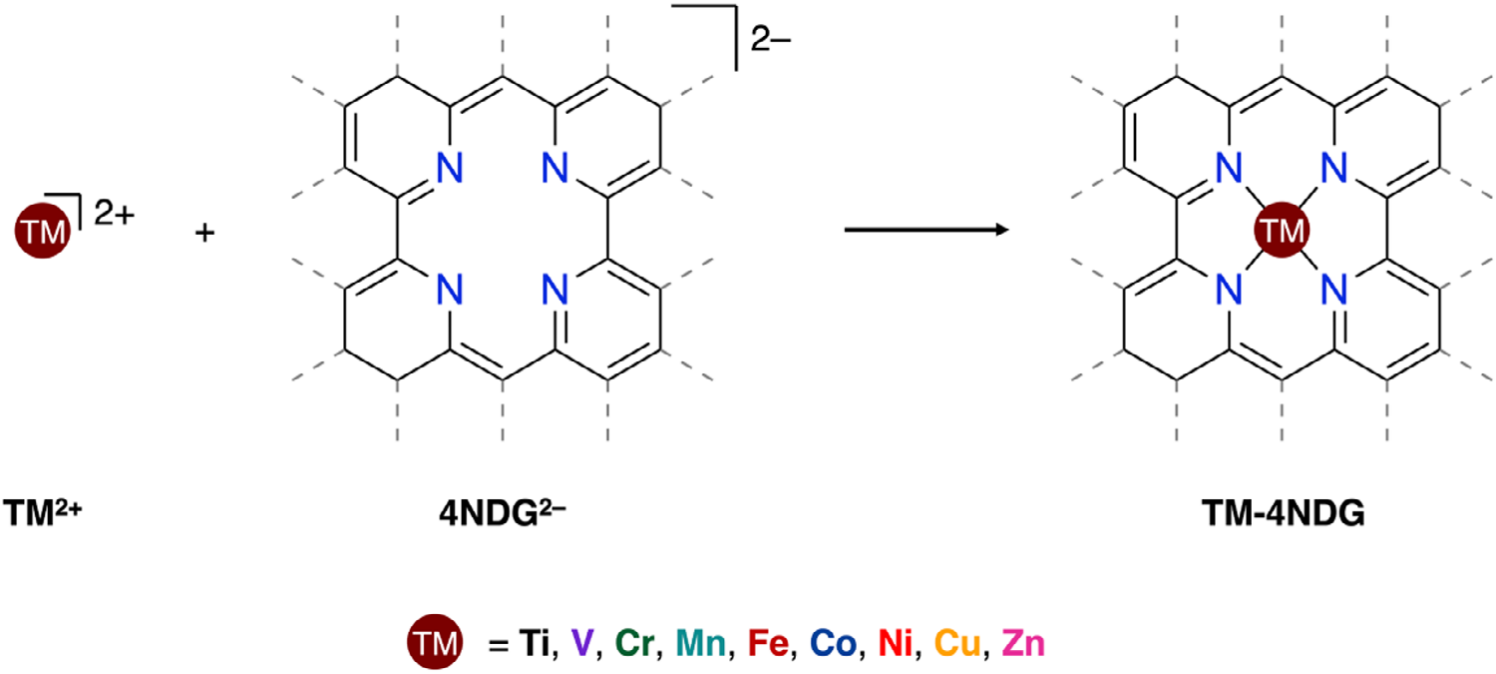
The bonding interaction in SACs between the TM (**TM^2+^**) and four‐nitrogen‐doped graphene support (**4NDG^2−^**), where TM = Ti, V, Cr, Mn, Fe, Co, Ni, Cu, and Zn.

In this work, we performed a detailed quantum chemical study to elucidate the nature and intrinsic stability of TM‐anchored nitrogen‐doped graphene SACs. To this end, we investigate the bonding mechanism, and trends therein between period 4 TM (TM^2+^) (TM = Ti, V, Cr, Mn, Fe, Co, Ni, Cu, and Zn) and four‐nitrogen‐doped graphene support (4NDG^2−^), using relativistic, dispersion‐corrected (periodic) density functional theory (DFT) at ZORA‐UPBE‐D3(BJ)/TZP (Scheme 1). Employing a reductionist approach, we analyze molecular single‐atom catalyst flakes, analogous to the SACs, using the activation strain model (ASM),^[^ ^14^ ^]^ Kohn‐Sham molecular orbital (KS‐MO)^[^ ^15^, ^16^ ^]^ theory, and canonical energy decomposition analysis (EDA)^[^ ^17^ ^]^ to gain quantitative insights into the origin of the bonding mechanism of TM‐4NDG SACs. This methodology has proven effective in elucidating fundamental aspects of chemical bonding and catalysis.^[^ ^18^, ^19^ ^]^

## 2 Computational Method

### 2.1 Computational Details

All spin‐polarized periodic DFT computations were performed using the BAND (BAND2023.104) module of the AMS software package.^[^ ^20^ ^]^ The generalized gradient approximation (GGA) functional PBE was used for the optimizations of all stationary points.^[^ ^21^ ^]^ The DFT‐D3(BJ) method developed by Grimme and coworkers,^[^ ^22^ ^]^ which contains the damping function proposed by Becke and Johnson,^[^ ^23^ ^]^ was used to describe nonlocal dispersion interactions. The basis set employed, denoted TZP,^[^ ^24^ ^]^ is of triple‐ζ quality and is augmented with one set of polarization functions on each atom. Scalar relativistic effects are accounted for using the zeroth‐order regular approximation (ZORA).^[^ ^25^ ^]^ This level of theory is referred to as ZORA‐UPBE‐D3(BJ)/TZP. To represent the single‐atom catalyst, we used a unit cell containing a single TM atom embedded in a 6 × 6 nitrogen‐doped graphene support. The *k*‐space was sampled using a regular grid with quality set to GOOD.^[^ ^26^ ^]^ The accuracies of the fit scheme (Zlm fit)^[^ ^26a^ ^]^ and the integration grid (Becke grid)^[^ ^26b^ ^]^ were set to GOOD. To obtain the SACs, we first optimized both the atomic positions and the lattice vectors of the nitrogen‐doped graphene support. After the addition of the TM, only the atomic positions were optimized while keeping the lattice vectors fixed. To calculate the bond energy (vide supra), we applied a neutralizing density for the charged fragments, that is, **TM^2+^** and **4NDG^2−^**.^[^ ^27^ ^]^

All model molecular single‐atom catalyst flakes, which comprise the BAND‐optimized structures with hydrogen added at the molecular boundary (see Figure S1 for a detailed description), were calculated using the ADF (ADF2023.104) module of the AMS software package.^[^ ^28^ ^]^ The ZORA‐UPBE‐D3(BJ)/TZP level of theory was used for all optimizations of stationary points and analysis. This level of theory at spin‐unrestricted formalism yields a nearly negligible spin contamination (see Tables S1 and S2).^[^ ^29^ ^]^ Additionally, single‐point energies were computed at ZORA‐UOPBE‐D3(BJ)/TZP^[^ ^30^ ^]^ on fully optimized ZORA‐UPBE‐D3(BJ)/TZP geometries to confirm bonding trends and spin states (see Tables S3, S4, and Figure S2). The **TM‐4NDG** bonding mechanism was evaluated as a function of the vertical displacement of **TM^2+^** with respect to nitrogen‐doped graphene support **4NDG** ^2−^ using the PyFrag 2019 program.^[^ ^31^ ^]^ Optimized structures were illustrated using CYLview.^[^ ^32^ ^]^

### 2.2 Activation Strain Model and Energy Decomposition Analysis

The bond energy in both the periodic (∆*E*_BAND_) and molecular systems (∆*E*_ADF_), is defined as the energy difference between the overall system and the individual fragments [Equation (1)]:

(1)

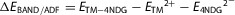




Here, *E*_TM‐4NDG_, *E*_TM_^2+^, and *E*_4NDG_^2−^ correspond to the energies of **TM‐4NDG**, **TM^2+^**, and **4NDG^2−^**, respectively, computed using either BAND or ADF.

Using the (periodic) activation strain model ((p)ASM)^[^ ^14^ ^]^ ∆*E* is decomposed into two components [Equation (2)]:

(2) $$\Delta E_{\mathrm{BAND}/\mathrm{ADF}}=\Delta E_{\mathrm{strain}}+\Delta E_{\mathrm{int}}$$

In this equation, the strain energy, Δ*E*_strain_, is the penalty that needs to be paid to deform the fragments from their equilibrium structure to the geometry they acquire in the overall system. On the other hand, the interaction energy, Δ*E*_int_, accounts for all the mutual interactions that occur between the geometrically deformed fragments in the overall system.

The interaction energy between the deformed fragments of the molecular system is further analyzed by means of our canonical energy decomposition analysis (EDA) scheme.^[^ ^17^ ^]^ The EDA decomposes the ∆*E*_int_ into the following four physically meaningful energy terms [Equation (3)]:

(3) $$\Delta E_{\mathrm{int}}=\Delta V_{\mathrm{elstat}}+\Delta E_{\mathrm{Pauli}}+\Delta E_{\mathrm{oi}}+\Delta E_{\mathrm{disp}}$$

Herein, Δ*V*_elstat_ is the classical electrostatic interaction between the unperturbed charge distributions of the (deformed) fragments. The Pauli repulsion, Δ*E*_Pauli_, comprises the destabilizing interaction between occupied closed‐shell orbitals of both fragments due to Pauli's exclusion principle. The orbital interaction energy, Δ*E*_oi_, accounts, that is, for one‐electron (e.g., SOMO–LUMO), electron‐pair (SOMO–SOMO), three‐electron bonding (e.g., SOMO–HOMO), polarization within fragments, and charge transfer between fragments (e.g., HOMO–LUMO). Nonlocal dispersion interactions ∆*E*_disp_ are described using the aforementioned D3(BJ) correction.^[^ ^22^ ^]^

The orbital interaction term (Δ*E*_oi_) can be further decomposed into the contributions stemming from each irreducible representation Γ of the point‐group symmetry of interacting fragments.^[^ ^33^ ^]^ For the analysis of the molecular systems, we use *C*_2v_ symmetry, which allows for the decomposition of Δ*E*_oi_ into four categories of orbital contributions corresponding to the irreducible representation of *C*_2v_ (for more details, see Figure S2) [Equation (4)]:

(4) $$\Delta E_{\mathrm{oi}}=\Delta E_{\mathrm{oi},A1}+\Delta E_{\mathrm{oi},A2}+\Delta E_{\mathrm{oi},B1}+\Delta E_{\mathrm{oi},B2}$$

## 3 Results and Discussion

### 3.1 Trends in TM‐4NDG Bond Strength

**Figure 1:**
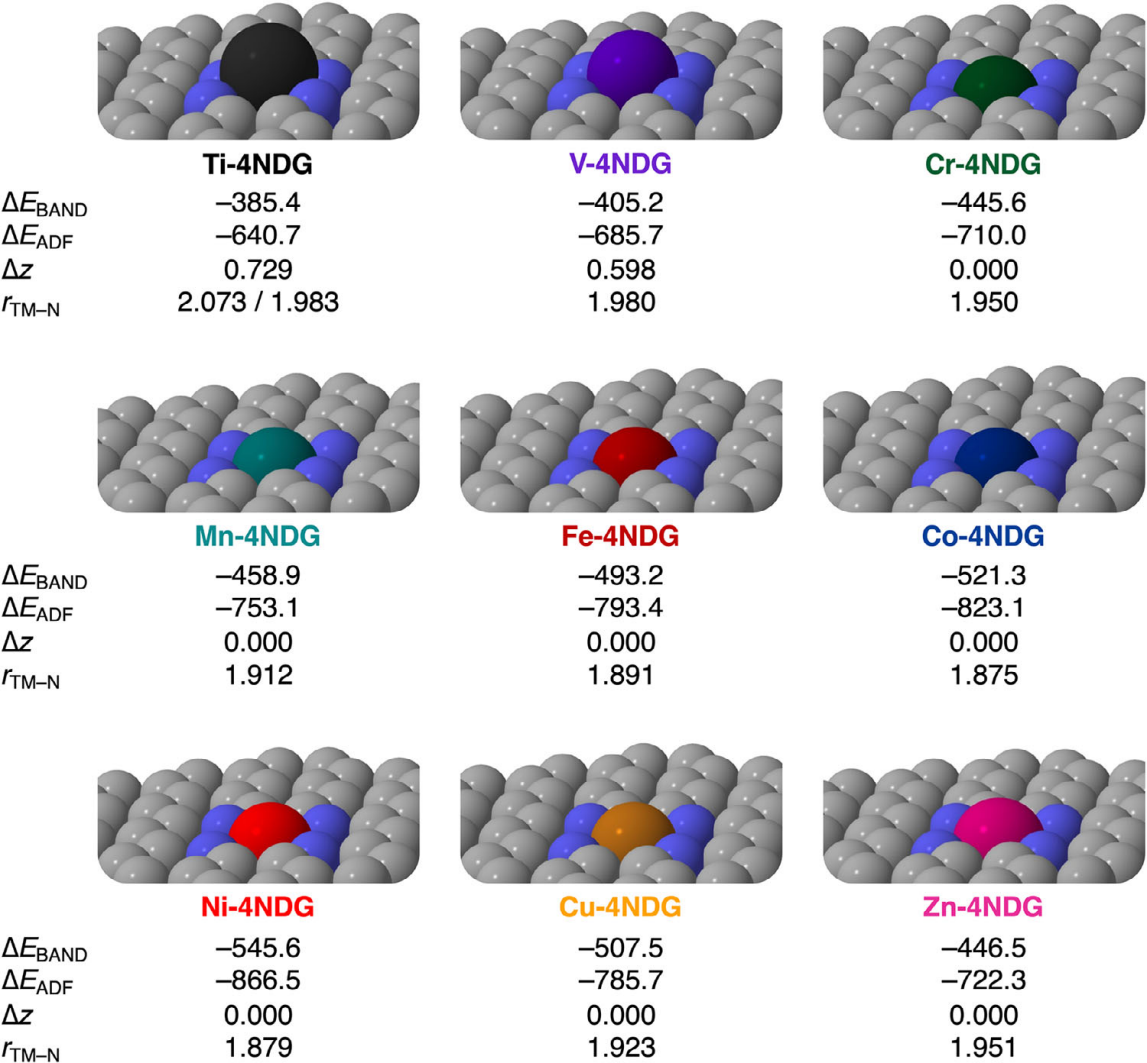
Equilibrium geometries of **TM‐4NDG** systems with their bond energies computed for the periodic systems (∆*E*_BAND_, in kcal mol^−1^) and molecular flake model systems (∆*E*_ADF_, in kcal mol^−1^), vertical displacement of **TM^2+^** with respect to nitrogen‐doped graphene support (∆*z*, in Å), and their distance to the neighboring nitrogen atoms (*r*_TM–N_, in Å), computed at ZORA‐UPBE‐D3(BJ)/TZP.

In transition metal‐anchored nitrogen‐doped graphene SACs (**TM‐4NDG**), the transition metal (**TM^2+^**) exhibits an oxidation state of 2+,^[^ ^4^, ^8^, ^13^, ^18^ ^]^ similar to TMs in metalloporphyrins.^[^ ^34^ ^]^ To accurately model the bonding interaction between the **TM^2+^** and nitrogen‐doped graphene (**4NDG^2−^**) and considering the correct oxidation states of the interacting fragments in the overall system, the **TM^2+^** fragment is assigned a charge of +2, while the support is assigned a charge of −2, resulting in an overall neutral system. Figure 1 shows the geometric and energetic data of the **TM‐4NDG** systems. The optimized geometries of the periodic **TM‐4NDG** systems are reported alongside their bond energies computed for the periodic systems, ∆*E*_BAND_, and the molecular flake model systems, ∆*E*_ADF_, the vertical displacement of **TM^2+^** with respect to nitrogen‐doped graphene support **4NDG^2−^**, ∆*z*, and the TM–N bond length in **TM‐4NDG**, *r*_TM–N_. The bond strength in the periodic **TM‐4NDG** systems between **TM^2+^** and **4NDG^2–^** becomes increasingly stabilizing along period 4 from **Ti‐4NDG** to **Ni‐4NDG**, going from −385.4 kcal mol^−1^ for **Ti‐4NDG** to −545.6 kcal mol^−1^ for **Ni‐4NDG**. However, proceeding further along period 4, an opposite trend is observed, namely, from **Ni‐4NDG** to **Zn‐4NDG**, the bond energy becomes less stabilizing, from −545.6 kcal mol^−1^ for **Ni‐4NDG** to −446.5 kcal mol^−1^ for **Zn‐4NDG**. For the molecular flakes model systems, the trend in bond strength is identical to the trend found for the periodic systems. Even though the trends in ∆*E*_BAND_ and ∆*E*_ADF_ are identical, the magnitudes somewhat differ as a result of the neutralizing density that has to be employed for the charged fragment in the periodic systems. For charged periodic systems a neutralizing density is required, as otherwise the Coulomb potential diverges.^[^ ^27^ ^]^ From **Ti‐4NDG** to **Ni‐4NDG**, the interaction systematically strengthens, while from **Ni‐4NDG** to **Zn‐4NDG,** it weakens. This highlights that the molecular flake model systems are well‐suited for describing the bonding situation in the periodic systems.

Examining the geometries of the **TM‐4NDG** systems, it is apparent that a shift in structure occurs from **Ti‐4NDG** to **Cr‐4NDG**. Where **Ti‐4NDG** and **V‐4NDG** are positioned above the graphene plane, ∆*z* = 0.729 Å and 0.598 Å, respectively, from **Cr‐4NDG** to **Zn‐4NDG**, all **TM^2+^** s lie in the graphene plane (∆*z* = 0.000 Å). A detailed description of the physical mechanism behind the vertical displacement of the **TM^2+^** will be provided later (see section: The origin of the vertical displacement). Furthermore, inspection of the geometries of the **TM‐4NDG** systems also reveals that the trend in TM–N bond distances generally correlates with the trend in bond energies, namely, when the bond becomes stronger, the bond distances decrease, and vice versa. Thus, from **Ti‐4NDG** to **Ni‐4NDG**, the bond energies become more stabilizing, and the TM–N bond distances shorten from 1.983 Å to 1.879 Å. The opposite is observed from **Ni‐4NDG** to **Zn‐4NDG**, where the bond energies become less stabilizing and the TM–N bond distances become longer from 1.879 Å to 1.951 Å. Notably, **Ti‐4NDG** is the only system in which the **TM^2+^** deviates from the center of the nitrogen‐doped graphene vacancy to maximize the bonding interaction, as evidenced by the unequal TM–N distances of 2.073 Å and 1.938 Å.

### 3.2 The Nature of the TM‐4NDG Bonding Interaction

To examine the origin of the trend in bond energies, we study the interaction between the **TM^2+^** and **4NDG^2−^** in more detail using the (periodic) activation strain model ((p)ASM).^[^ ^14^ ^]^ For both the periodic systems and molecular flake model systems, we find that the trend in bond energies is entirely governed by the interaction energy (Tables S5 and S6). In the periodic systems, the interaction energies become more stabilizing from **Ti‐4NDG** to **Ni‐4NDG**, ranging from −403.0 to −554.5 kcal mol^−1^ (and from −653.2 to −875.2 kcal mol^−1^ in the molecular flake model systems), and less stabilizing from **Ni‐4NDG** to **Zn‐4NDG,** from −554.5 to −454.9 kcal mol^−1^ (−875.2 to −730.1 kcal mol^−1^ for the molecular flake model systems). The strain energy, on the other hand, remains nearly constant for all systems and hence does not contribute to the observed trend. This further corroborates that the molecular flakes are good model systems for understanding the bonding interaction between **TM^2+^** and **4NDG^2−^** in the periodic SACs.

**Figure 2:**
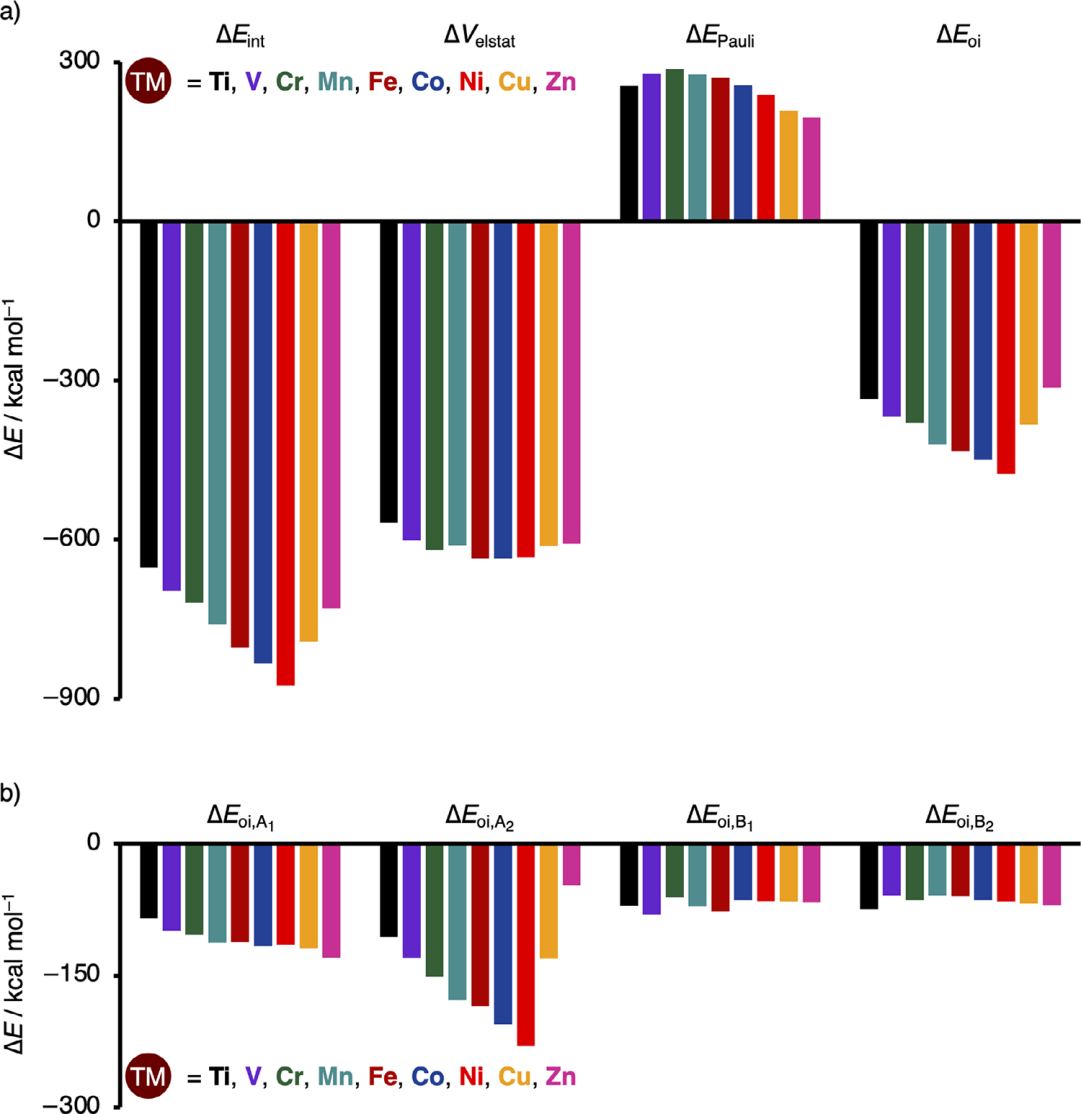
a) Energy decomposition analysis (in kcal mol^−1^), and b) decomposition of orbital interaction energy Δ*E*_oi_ (in kcal mol^−1^) for the interaction between the transition metal (**TM^2+^**) and nitrogen‐doped graphene support (**4NDG**) in **TM‐4NDG**, where TM = Ti, V, Cr, Mn, Fe, Co, Ni, Cu, and Zn, computed at ZORA‐UPBE‐D3(BJ)/TZP. See Tables S7 and S8 for numerical data.

To elucidate the trend in interaction energy, we further decompose the interaction energy of the molecular flake model system using the energy decomposition analysis (EDA).^[^ ^17^ ^]^ We find that the trend in interaction energy primarily arises from the orbital interactions (Figure 2a). Resembling the trend in bond and interaction energy, the orbital interactions become increasingly stabilizing from **Ti‐4NDG** to **Ni‐4NDG**, ranging from −335.3 to −475.9 kcal mol^−1^, and drop down in stability from **Ni‐4NDG** to **Zn‐4NDG** from −475.9 to −313.2 kcal mol^−1^. The electrostatic interaction and Pauli repulsion, on the other hand, do not contribute to the observed trend. Where the electrostatic interaction exhibits no clear trend, the Pauli repulsion shows, in fact, an opposite trend from **Ti‐4NDG** to **Cr‐4NDG**, becoming more destabilizing and from **Cr‐4NDG** to **Zn‐4NDG** less destabilizing.

Because our molecular single‐atom catalyst flakes belong to the *C*_2v_ point group, we can decompose the orbital interactions even further into the contribution stemming from the irreps of *C*_2v_, which allows us to obtain a more detailed insight into the bonding mechanism responsible for the observed trend in bond strength (Figure 2a). The orbital interactions primarily arise from the interaction in the A_2_ irrep, which for the **TM^2+^** comprises the 3*d*_xy_ atomic orbital and the nitrogen lone pair orbitals of the support (Figure S2). As the bond becomes stronger from **Ti‐4NDG** to **Ni‐4NDG**, the interactions in the A_2_ irrep increase from −106.5 to −230.4 kcal mol^−1^. As the bonding interactions weaken from **Ni‐4NDG** to **Zn‐4NDG**, so does the interaction in the A_2_ irrep, declining in stabilization from −230.4 to −47.4 kcal mol^−1^. In contrast, the other irreps, that is, A_1_, B_1_, and B_2_, show little variation in interaction strength and hence no clear trends. Notably, these observations are further confirmed using the Voronoi deformation density charge analysis (Table S9).^[^ ^35^ ^]^ Therefore, the interaction in the A_2_ irrep is essential for the observed trend in bond strength upon changing the **TM^2+^** along period 4.

**Figure 3:**
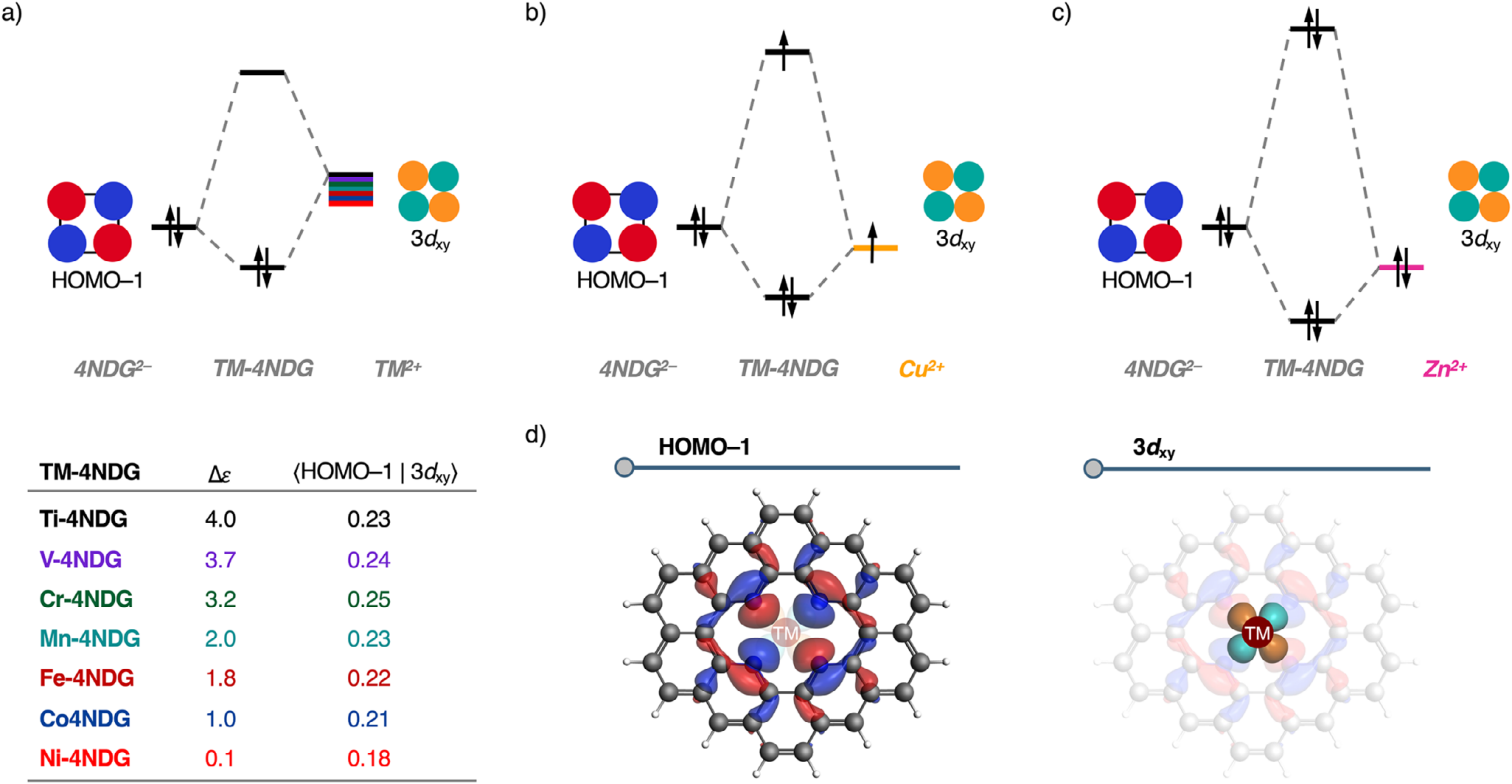
a) Schematic molecular orbital (MO) diagram and the overlaps and energy gaps (β‐spin, in eV) of the HOMO–LUMO interaction between period 4 **TM^2+^** and nitrogen‐doped graphene **4NDG^2−^**; schematic MO diagram of b) the HOMO–SOMO interaction between **4NDG^2−^** and **Cu^2+^**; c) the HOMO–HOMO interaction between **4NDG^2−^** and **Zn^2+^** and d) key orbitals of **4NDG^2−^** and **TM^2+^** (isovalue = 0.03 Bohr^−3/2^), computed at ZORA‐UPBE‐D3(BJ)/TZP (for α‐spin, see Figure S5).

The origin of the trend in orbital interaction in the A_2_ irrep is further investigated by performing a Kohn‐Sham molecular orbital (KS‐MO) analysis (Figure 3).^[^ ^15^, ^16^ ^]^ From **Ti‐4NDG** to **Ni‐4NDG,** the more stabilizing orbital interaction originates from a consistently more stabilizing HOMO–LUMO interaction, due to the systematically smaller HOMO–LUMO energy gap. The nature of this bonding mechanism changes for **Cu‐4NDG** and **Zn‐4NDG**, where it becomes a less favorable HOMO–SOMO^[^ ^36^ ^]^ and even unfavorable HOMO–HOMO interaction, respectively, resulting in a weakening of the orbital interaction. The key orbitals involved in the interaction in the A_2_ irrep are the HOMO–1 of the **4NDG^2−^** fragment and the 3*d*_xy_ atomic orbital of the **TM^2+^** fragment. The HOMO–1 of **4NDG^2−^** contains the lone pairs of the nitrogen atoms with two nodal planes, pointing toward the lobes of the 3*d*_xy_ atomic orbital (Figure 3d). From **Ti‐4NDG** to **Ni‐4NDG**, the interaction between the filled HOMO–1 and the empty 3*d*_xy_ is a HOMO–LUMO type interaction, for which the orbital interaction energy is proportional to the HOMO–LUMO overlap *S* squared divided by its energy gap Δε, *S* ^2^/Δε (Figure 3a).^[^ ^16^ ^]^ The increasingly stabilizing character of this interaction originates from the decreasing HOMO–LUMO energy gap, which, from **Ti‐4NDG** to **Ni‐4NDG**, decreases from 4.0 eV to 0.1 eV. This decrease in HOMO–LUMO energy gap can be attributed to the increased effective nuclear charge of **TM^2+^**. As the effective nuclear charge on the **TM^2+^** becomes larger along period 4, the orbitals of the **TM^2+^** become systematically more stabilized, resulting in a smaller HOMO–LUMO energy gap and hence stronger orbital interactions. Conversely, the HOMO–LUMO overlap generally decreases from **Ti‐4NDG** to **Ni‐4NDG**, which is also a consequence of the increased effective nuclear charge. As the effective nuclear charge of **TM^2+^** increases, the electrons are pulled closer to the nucleus, resulting in spatially smaller orbitals and hence reduced orbital overlaps (Figure S4).

The picture changes when we proceed from **Ni‐4NDG** to **Cu‐4NDG** to **Zn‐4NDG**, because of the increasing population of the **TM^2+^**’s 3*d*_xy_ atomic orbital (Table S10). For **Cu‐4NDG**, the 3*d*_xy_ is singly occupied, making the interaction between the HOMO–1 of **4NDG^2−^** and the 3*d*_xy_ of **Cu^2+^** a HOMO–SOMO interaction (Figure 3b). This type of orbital interaction is intrinsically less stabilizing than a HOMO–LUMO interaction, due to its partially repulsive nature,^[^ ^36^ ^]^ leading to a loss of stabilizing orbital interaction, and hence bond energy, from **Ni‐4NDG** to **Cu‐4NDG**. This effect becomes even more pronounced for **Zn‐4NDG**, where the 3*d*_xy_ orbital becomes doubly occupied. As a consequence, the interaction becomes even less stabilizing, causing the overall interaction in the A_2_ irrep to become significantly smaller (Figure 3c). Thus, the **TM**‐**4NDG** bond becomes more stabilizing from **Ti‐4NDG** to **Ni‐4NDG**, due to an enhanced HOMO–LUMO interaction as a result of the increasing effective nuclear charge. However, this bond becomes weaker from **Ni‐4NDG** to **Zn‐4NDG**, because the increasing filling of the 3*d*_xy_ atomic orbital of **TM^2+^** changes the bonding mechanism from a favorable HOMO–LUMO to a less favorable HOMO–SOMO to an unfavorable HOMO–HOMO interaction.

### 3.3 The Origin of the Vertical Displacement in Ti‐4NDG and V‐4NDG

**Figure 4:**
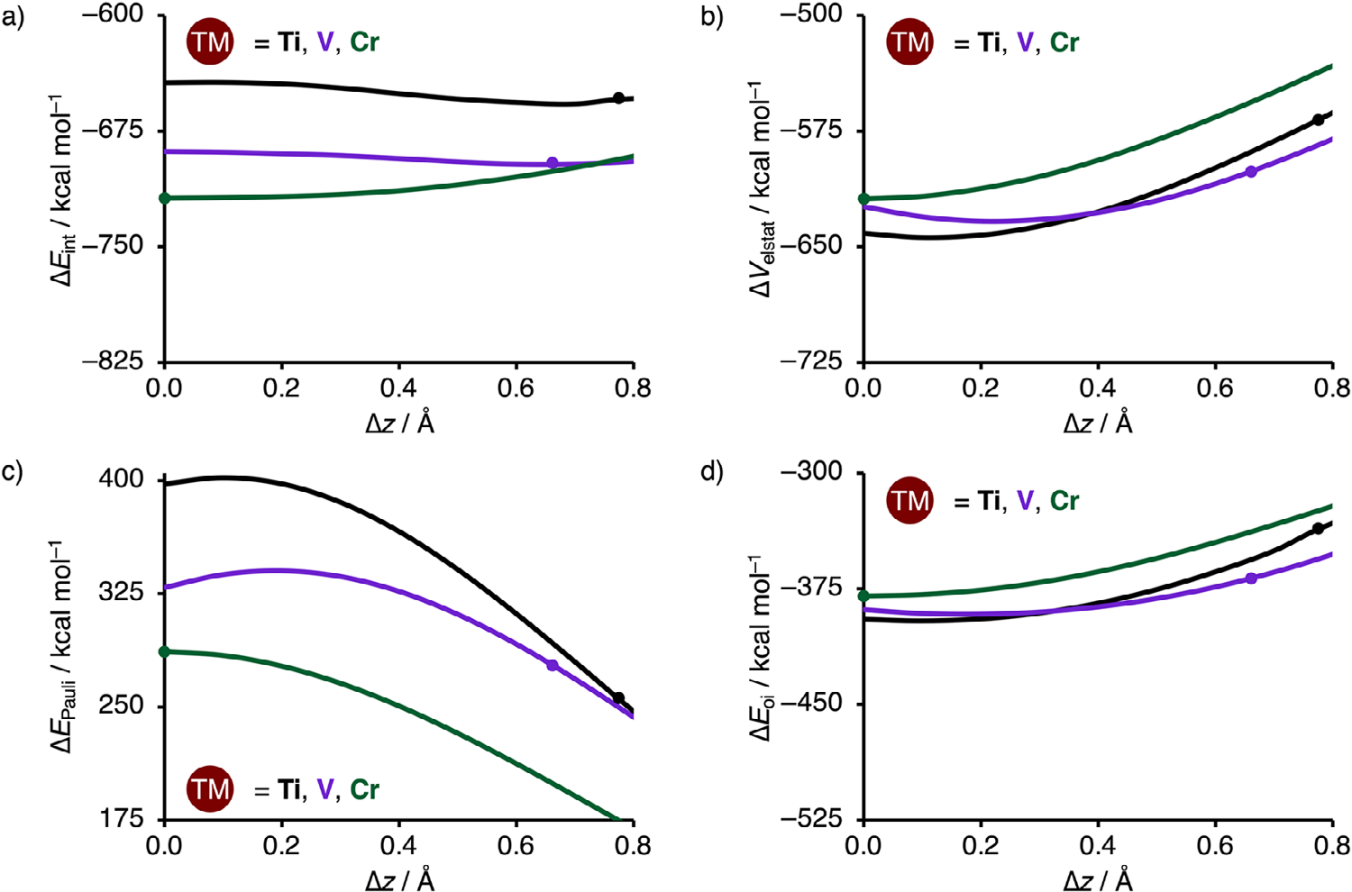
Energy decomposition analysis (in kcal mol^−1^): a) interaction energy; b) electrostatic interaction; c) Pauli repulsion; and d) orbital interaction of the **TM^2+^⋅⋅⋅4NDG^2−^** bond (with TM = Ti, V, and Cr) as a function of the vertical displacement of **TM^2+^** with respect to **4NDG^2−^**, computed at ZORA‐UPBE‐D3(BJ)/TZP. The equilibrium **TM‐4NDG** distances are indicated by a dot.

In this section, we want to address why for **Ti‐4NDG** and **V‐4NDG** the **TM^2+^** lies above the **4NDG^2−^** plane, while for **Cr‐4NDG** to **Zn‐4NDG** the **TM^2+^** lies in the plane of **4NDG^2−^** (Figure 1). To this end, we have analyzed and compared **Ti‐4NDG**, **V‐4NDG**, and **Cr‐4NDG** using the EDA as a function of the vertical displacement of **TM^2+^** with respect to **4NDG^2−^**, ∆*z* (Figure 4). Note that the EDA diagram containing all systems can be found in Figure S6. Our analysis reveals that, in line with the results in Figure 1, **Ti‐4NDG** and **V‐4NDG** have the most stabilizing interaction energy when the respective **Ti^2+^** and **V^2+^** lie above the plane of **4NDG^2−^**, whereas **Cr‐4NDG** has the most favorable interaction energy when **Cr^2+^** is in the plane of **4NDG^2−^** (Figure 4a). This difference is exclusively determined by the larger magnitude and steeper rise of the repulsive Pauli repulsion for **Ti‐4NDG** and **V‐4NDG** compared to **Cr‐4NDG** (Figure 4c). In contrast, the attractive electrostatic and orbital interactions are for all systems more stabilizing when **TM^2+^** lies in the plane of **4NDG^2−^** (Figure 4b,d). Ultimately, it is the balance between the buildup of destabilizing Pauli repulsion (pushing the **TM^2+^** out of the **4NDG^2−^** plane) and the stabilizing electrostatic and orbital interactions (pulling the **TM^2+^** into the **4NDG^2−^** plane) that determines the position of the **TM^2+^** with respect to the plane of **4NDG^2−^**. In the case of **Ti‐4NDG** and **V‐4NDG**, this pulling effect of the electrostatic and orbital interactions is insufficient to overcome the strong pushing effect of repulsive Pauli repulsion at short ∆*z* distances. As a result, **Ti^2+^** and **V^2+^** are positioned above the **4NDG^2−^** plane. For **Cr^2+^**, on the other hand, the Pauli repulsion is significantly less stabilizing, allowing the stabilizing electrostatic and orbital interactions to pull **Cr^2+^** into the **4NDG^2−^** plane.

**Figure 5:**
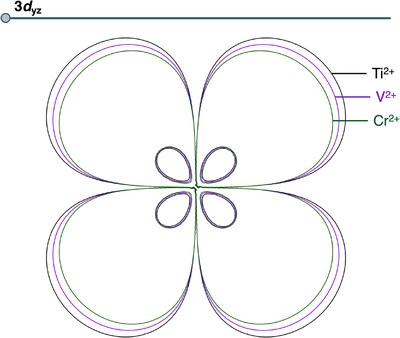
Contour plot of the atomic 3*d*_yz_ orbitals of the **TM^2+^** fragment with **TM^2+^** = **Ti^2+^** (black), **V^2+^** (purple), and **Cr^2+^** (green). All contour plots contain 2 contours from 0.0195 – 0.4000 au, computed at ZORA‐UPBE‐D3(BJ)/TZP.

The origin of the enhanced and steeply rising Pauli repulsion for **Ti‐4NDG** and **V‐4NDG** compared to **Cr‐4NDG** can be traced back to the spatial extent of the atomic orbitals of the **TM^2+^** s along period 4. In Figure 5, we have visualized the 3*d*_yz_ atomic orbital of **Ti^2+^**, **V^2+^**, and **Cr^2+^** as an illustrative example of their differences in effective size (see Figure S4 for all period 4 TMs). From **Ti** to **Cr**, the size of the atomic orbital decreases, which reduces the repulsive occupied–occupied orbital overlap between the occupied orbitals of **TM^2+^** and **4NDG^2−^** and hence reduces the repulsive Pauli repulsion when these fragments approach each other. The reduced atomic orbital size along period 4 is a result of the increasing effective nuclear charge of the **TM^2+^**. As the effective nuclear charge increases, the electrons are pulled more strongly to the nucleus, resulting in spatially smaller orbitals and hence less repulsive occupied–occupied orbital overlap. Thus, for **Ti‐4NDG** and **V‐4NDG**, the atomic orbitals of **Ti^2+^** and **V^2+^**, respectively, are effectively too large and, therefore, build up too much repulsion to reside in the plane of the **4NDG**. Going further along period 4, this effect is diminished, giving rise to structures where the **TM^2+^** is positioned in the plane of **4NDG**.

## 4 Conclusion

In this work, we have investigated the nature and stability of period 4 TM‐anchored nitrogen‐doped graphene SACs (**TM‐4NDG**, where TM = Ti, V, Cr, Mn, Fe, Co, Ni, Cu, and Zn). By studying the bonding interaction between the period 4 TM and nitrogen‐doped graphene support, we find that this bond strengthens from **Ti‐4NDG** to **Ni‐4NDG**, but weakens when we move further along period 4, from **Ni‐4NDG** to **Zn‐4NDG**. Furthermore, **Ti‐4NDG** and **V‐4NDG** have optimal geometries when the **TM^2+^** is positioned above the plane of nitrogen‐doped graphene, whereas from **Cr‐4NDG** to **Zn‐4NDG**, the **TM^2+^** always lies in the graphene plane. This follows from our quantum chemical (periodic) activation strain model ((p)ASM) and quantitative molecular orbital (MO analyses based on relativistic, dispersion‐corrected (periodic) DFT at ZORA‐UPBE‐D3(BJ)/TZ2P.

Our analyses reveal that the trend in the bonding interaction between **TM^2+^** and **4NDG^2−^** along period 4 depends on two intrinsic features of the **TM^2+^**: (i) the larger effective nuclear charge of **TM^2+^**; and (ii) the increasing filling of the *d*‐atomic orbitals of **TM^2+^**. The major bonding mechanism is the interaction between the 3*d*_xy_ atomic orbital of the **TM^2+^** and the nitrogen lone pair orbitals **4NDG^2−^**. From **Ti‐4NDG** to **Ni‐4NDG**, the increasing effective nuclear charge of **TM^2+^** lowers the 3*d*_xy_ atomic orbital of the **TM^2+^** in energy, leading to a smaller HOMO–LUMO gap and hence stronger interaction. Proceeding to **Cu‐4NDG** and **Zn‐4NDG**, the bonding interaction becomes weaker, because the 3*d*_xy_ atomic orbital of the **TM^2+^** becomes singly and doubly occupied, thereby changing the bonding mechanism from a favorable HOMO–LUMO to a less favorable HOMO–SOMO to an unfavorable HOMO–HOMO interaction.

The elevation of the early period 4 TMs, that is, **Ti^2+^** and **V^2+^**, above the plane of **4NDG^2−^** originates from the significantly more destabilizing Pauli repulsion they build up upon approaching **4NDG^2−^** compared to the later **TM^2+^** s. This increased destabilizing Pauli repulsion for **Ti‐4NDG** and **V‐4NDG** can be traced back to the large effective size of the *d*‐atomic orbitals of **Ti^2+^** and **V^2+^**. The large *d*‐atomic orbitals observed for the early **TM^2+^** s in period 4 are a result of their lower nuclear charge, resulting in a reduced electron–nucleus attraction and hence spatially larger atomic orbitals, which, in turn, engage in more destabilizing Pauli repulsion with **4NDG^2−^**. From **Cr‐4NDG** to **Zn‐4NDG**, the higher effective nuclear charge of the **TM^2+^** reduces the effective size of the *d*‐atomic orbitals to such an extent that the buildup of destabilizing Pauli repulsion is significantly smaller, allowing them to position in the plane of the **4NDG^2−^**. We envision that the insights obtained in this work can aid in the design of novel SACs.

## Conflict of Interest

There are no conflicts to declare.

## Supporting information



Supporting Information

## Data Availability

The data that supports the findings of this study are available within the article and its supplementary material. Cartesian coordinates can be downloaded from Yoda VU, see Ref. [37].
