# Supplementary material for: The Nature and Stability of Transition Metal‐Anchored Nitrogen‐Doped Graphene Single‐Atom Catalysts
Source: Chemistry. 2025 Jul 14;31(43):e202501654. doi: 10.1002/chem.202501654 (PMC12319401; doi:10.1002/chem.202501654)
Supplement: Supplementary file 1 — Supporting Information [file CHEM-31-e202501654-s001.pdf]

Content

Figure S1. Schematic representation of computational method for deriving the molecular single-atom catalyst flake from the periodic structure. Color code of the ball structures: hydrogen = white; carbon = gray; nitrogen = blue; TM = dark red.

Table S1. All spin states of the pseudo square planar complex **TM-4NDG** systems evaluated in this work, where the most stable spin state is highlighted in **bold**. The expectation value of the s^2 operator ($\langle s^2 \rangle$) is shown in between brackets.

Table S2. All spin states of the pseudo square planar complex **TM-4NDG** systems evaluated in this work, where the most stable spin state is highlighted in **bold**. The electronic energies of the **TM-4NDG** systems are shown below the spin states (in kcal mol^{-1}).

Table S3. All spin states of the pseudo square planar complex **TM-4NDG** systems evaluated in this work, where the most stable spin state is highlighted in **bold**. The expectation value of the s^2 operator ($\langle s^2 \rangle$) is shown in between brackets, computed at ZORA-UOPBE-D3(BJ)/TZP.

Table S4. All spin states of the pseudo square planar complex **TM-4NDG** systems evaluated in this work, where the most stable spin state is highlighted in **bold**. The electronic energies of the **TM-4NDG** systems are shown below the spin states (in kcal mol^{-1}), computed at ZORA-UOPBE-D3(BJ)/TZP.

Figure S2. Energy decomposition analysis (in kcal mol^{-1}) for the interaction between the transition metal (**TM^{2+}**) and nitrogen-doped graphene support (**4NDG^{2-}**) in **TM-4NDG**, where TM = Ti, V, Cr, Mn, Fe, Co, Ni, Cu, and Zn, computed at a) ZORA-UPBE-D3(BJ)/TZP b) ZORA-UOPBE-D3(BJ)/TZP.

Figure S3. Representation of fragment molecular orbitals of **TM^{2+}** and **4NDG^{2-}** (isovalue = 0.03 Bohr$^{-3/2}$), sorted by the irreducible representations of the C_{2v} point group and computed at ZORA-UPBE-D3(BJ)/TZP.

Table S5. Periodic activation strain analyses (in kcal mol^{-1}) for the interaction between the transition metal (**TM^{2+}**) and nitrogen-doped graphene support (**4NDG**) in **TM-4NDG**.

Table S6. Activation strain analyses (in kcal mol^{-1}) for the interaction between the transition metal (**TM^{2+}**) and nitrogen-doped graphene support (**4NDG**) in **TM-4NDG**.

Table S7. Energy decomposition analysis (in kcal mol^{-1}) for the interaction between the transition metal (**TM^{2+}**) and nitrogen-doped graphene support (**4NDG**) in **TM-4NDG**.

Table S8. Orbital interaction energies decomposed into irreducible representation of C_{2v} (in kcal mol^{-1}) for the interaction between the transition metal (**TM^{2+}**) and nitrogen-doped graphene support (**4NDG**) in **TM-4NDG**.

Table S9. Change in Voronoi deformation density (VDD) atomic charge ΔQ (in electrons) due to the formation of the bond between the **TM^{2+}** and **4NDG^{2-}**, decomposed into contributions stemming from Pauli repulsion and orbital interactions ($\Delta Q = \Delta Q_{Pauli} + \Delta Q_{oi}$) and into contributions from the various irreps (Γ) of the molecular point-group symmetry (C_{2v}).

Figure S4. Contour plot of the atomic $3d_{xy}$ orbitals of the **TM^{2+}** fragment with **TM^{2+}**= **Ti^{2+}** (black), **V^{2+}** (purple), **Cr^{2+}** (green), **Mn^{2+}** (turquoise), **Fe^{2+}** (dark red), **Co^{2+}** (blue), **Ni^{2+}** (bright red), **Cu^{2+}** (orange), and **Zn^{2+}** (pink). All contour plots contain 2 contours from 0.0195 – 0.4000 au, computed at ZORA-UPBE-D3(BJ)/TZP.

Figure S5. a) Schematic molecular orbital diagram and the overlaps and energy gaps (α-spin, in eV) of the HOMO–LUMO interaction between period 4 transition metal **TM^{2+}** and nitrogen-doped graphene **4NDG^{2-}**; Schematic molecular orbital diagram of b) the HOMO–SOMO interaction between **4NDG^{2-}** and **Cu^{2+}**; c) the HOMO–HOMO interaction between **4NDG^{2-}** and **Zn^{2+}** and d) key orbitals of **4NDG^{2-}** and **TM^{2+}** (isovalue = 0.03 Bohr$^{-3/2}$), computed at ZORA-UPBE-D3(BJ)/TZP.

Table S10. **TM^{2+}** and **4NDG^{2-}** ground state occupations and valence occupations expressed in irreducible representations of C_{2V}.

Figure S6. Energy decomposition analysis (in kcal mol^{-1}): a) interaction energy; b) electrostatic interaction; c) Pauli repulsion; and d) orbital interaction of the **TM^{2+}···4NDG^{2-}** bond (with **TM^{2+}** = Ti, V, Cr, Mn, Fe, Co, Ni, Cu, and Zn) as function of the vertical displacement of **TM^{2+}** with respect to **4NDG^{2-}**, computed at ZORA-UPBE-D3(BJ)/TZP.

Table S11. Energy decomposition analysis (in kcal mol^{-1}) for the interaction between the transition metal (**TM^{2+}**) and nitrogen-doped graphene support (**4NDG**) in **TM-4NDG**, computed at ZORA-UOPBE-D3(BJ)/TZP.

Table S12. Orbital interaction energies decomposed into irreducible representations of C_{2V} (in kcal mol^{-1}) for the interaction between the transition metal (**TM^{2+}**) and nitrogen-doped graphene support (**4NDG**) in **TM-4NDG**, computed at ZORA-UOPBE-D3(BJ)/TZP.

Table S13. Total energies of the **TM-4NDG** systems (in kcal mol^{-1}) optimized using C_1 and C_{2v} symmetry.

Table S14. Total energies of the **TM-4NDG** systems (in kcal mol^{-1}) optimized using periodic DFT (BAND) and DFT (ADF) with C_1 and C_{2v} symmetry.

Table S15. Voronoi deformation density (VDD) atomic charge Q (in electrons) on the transition metal in **TM-4NDG** optimized using periodic DFT (BAND).

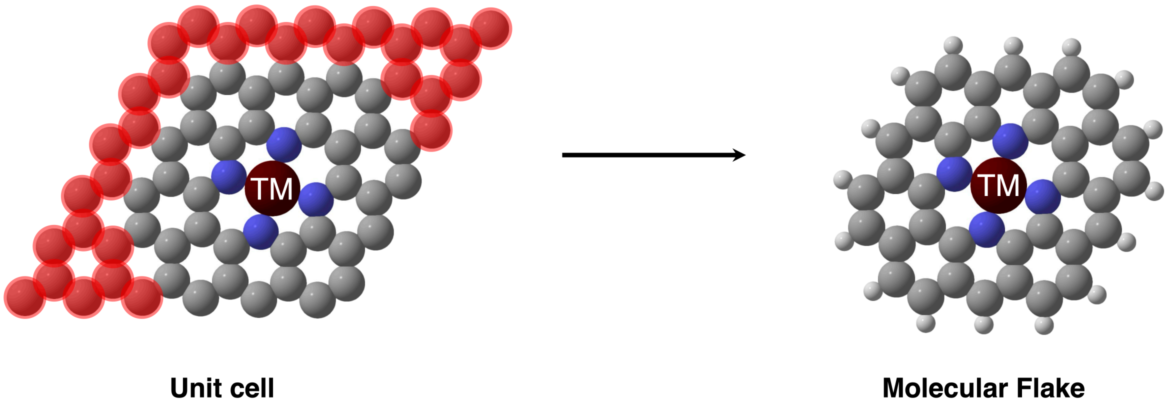

Figure S1. Schematic representation of computational method for deriving the molecular single-atom catalyst flake from the periodic structure. Color code of the ball structures: hydrogen = white; carbon = gray; nitrogen = blue; TM = dark red.

In our computational approach, the characteristics of the transition metal centers, as found in the periodic systems, are preserved in molecular single-atom catalyst flakes. First, the BAND-optimized coordinates are imported into ADF. From the BAND unit cell, the carbon atoms beyond the second coordination sphere surrounding the catalytically active center are removed, that is, all carbon atoms marked by red spheres in Figure S1. Since our system now has dangling bonds, the molecular boundary is capped with hydrogen atoms. The C–H bonds are optimized while all other coordinates remain frozen. Furthermore, for the detailed activation strain and energy decomposition analysis, the molecular single-atom catalyst flakes are converted to C_{2v} symmetry at a nearly negligible energetic cost (see Table S13).

Table S1. All spin states of the pseudo square planar complex **TM-4NDG** systems evaluated in this work, where the most stable spin state is highlighted in **bold**. The expectation value of the s^2 operator ($\langle s^2 \rangle$) is shown in between brackets.[a]

TM-4NDG	Number of *d*-electrons	Low	Intermediate	High
**Ti-4NDG**	2	Singlet (0.00)	**Triplet** (2.01)	–
**V-4NDG**	3	Doublet[b]	**Quartet** (3.77)	–
**Cr-4NDG**	4	Singlet[b]	Triplet[b]	**Quintet** (6.02)
**Mn-4NDG**	5	Doublet[b]	**Quartet** (3.89)	Sextet (8.85)
**Fe-4NDG**	6	Singlet[b]	**Triplet** (2.03)	–
**Co-4NDG**	7	**Doublet** (0.76)	Quartet (3.77)	–
**Ni-4NDG**	8	**Singlet** (0.00)	Triplet (2.01)	–
**Cu-4NDG**	9	**Doublet** (0.75)	–	–
**Zn-4NDG**	10	**Singlet** (0.00)	–	–

[a] Computed at ZORA-UPBE-D3(BJ)/TZP. [b] Electronic structure does not exist.

Table S2. All spin states of the pseudo square planar complex **TM-4NDG** systems evaluated in this work, where the most stable spin state is highlighted in **bold**. The electronic energies of the **TM-4NDG** systems are shown below the spin states (in kcal mol^{-1}).[a]

TM-4NDG	Number of *d*-electrons	Low	Intermediate	High
**Ti-4NDG**	2	Singlet<br>–9769.3	**Triplet**<br>–9773.6	–
**V-4NDG**	3	Doublet[b]	**Quartet**<br>–9810.2	–
**Cr-4NDG**	4	Singlet[b]	Triplet[b]	**Quintet**<br>–9854.1
**Mn-4NDG**	5	Doublet[b]	**Quartet**<br>–9848.5	Sextet<br>–9833.3
**Fe-4NDG**	6	Singlet[b]	**Triplet**<br>–9834.2	–
**Co-4NDG**	7	**Doublet**<br>–9816.4	Quartet<br>–9802.9	–
**Ni-4NDG**	8	**Singlet**<br>–9765.3	Triplet<br>–9750.2	–
**Cu-4NDG**	9	**Doublet**<br>–9695.6	–	–
**Zn-4NDG**	10	**Singlet**<br>–9649.5	–	–

[a] Computed at ZORA-UPBE-D3(BJ)/TZP. [b] Electronic structure does not exist.

Table S3. All spin states of the pseudo square planar complex **TM-4NDG** systems evaluated in this work, where the most stable spin state is highlighted in **bold**. The expectation value of the s^2 operator ($\langle s^2 \rangle$) is shown in between brackets, computed at ZORA-UOPBE-D3(BJ)/TZP.

TM-4NDG	Number of *d*-electrons	Low	Intermediate	High
**Ti-4NDG**	2	Singlet[a]	**Triplet** (2.02)	–
**V-4NDG**	3	Doublet[a]	**Quartet** (4.02)	–
**Cr-4NDG**	4	Singlet[a]	Triplet[a]	**Quintet** (6.04)
**Mn-4NDG**	5	Doublet[a]	**Quartet** (3.99)	Sextet (8.95)
**Fe-4NDG**	6	Singlet[a]	**Triplet** (2.03)	–
**Co-4NDG**	7	**Doublet** (0.77)	Quartet (3.77)	–
**Ni-4NDG**	8	**Singlet** (0.00)	Triplet (2.02)	–
**Cu-4NDG**	9	**Doublet** (0.75)	–	–
**Zn-4NDG**	10	**Singlet** (0.00)	–	–

[a] Electronic structure does not exist.

Table S4. All spin states of the pseudo square planar complex **TM-4NDG** systems evaluated in this work, where the most stable spin state is highlighted in **bold**. The electronic energies of the **TM-4NDG** systems are shown below the spin states (in kcal mol^{-1}), computed at ZORA-UOPBE-D3(BJ)/TZP.

TM-4NDG	Number of *d*-electrons	Low	Intermediate	High
**Ti-4NDG**	2	Singlet[a]	**Triplet**<br>–10039.2	–
**V-4NDG**	3	Doublet[a]	**Quartet**<br>–10071.3	–
**Cr-4NDG**	4	Singlet[a]	Triplet[a]	**Quintet**<br>–10120.9
**Mn-4NDG**	5	Doublet[a]	**Quartet**<br>–10115.8	Sextet<br>–10100.8
**Fe-4NDG**	6	Singlet[a]	**Triplet**<br>–10095.6	–
**Co-4NDG**	7	**Doublet**<br>–10072.8	Quartet<br>–10058.1	–
**Ni-4NDG**	8	**Singlet**<br>–10007.8	Triplet<br>–9993.4	–
**Cu-4NDG**	9	**Doublet**<br>–9940.4	–	–
**Zn-4NDG**	10	**Singlet**<br>–9902.6	–	–

[a] Electronic structure does not exist.

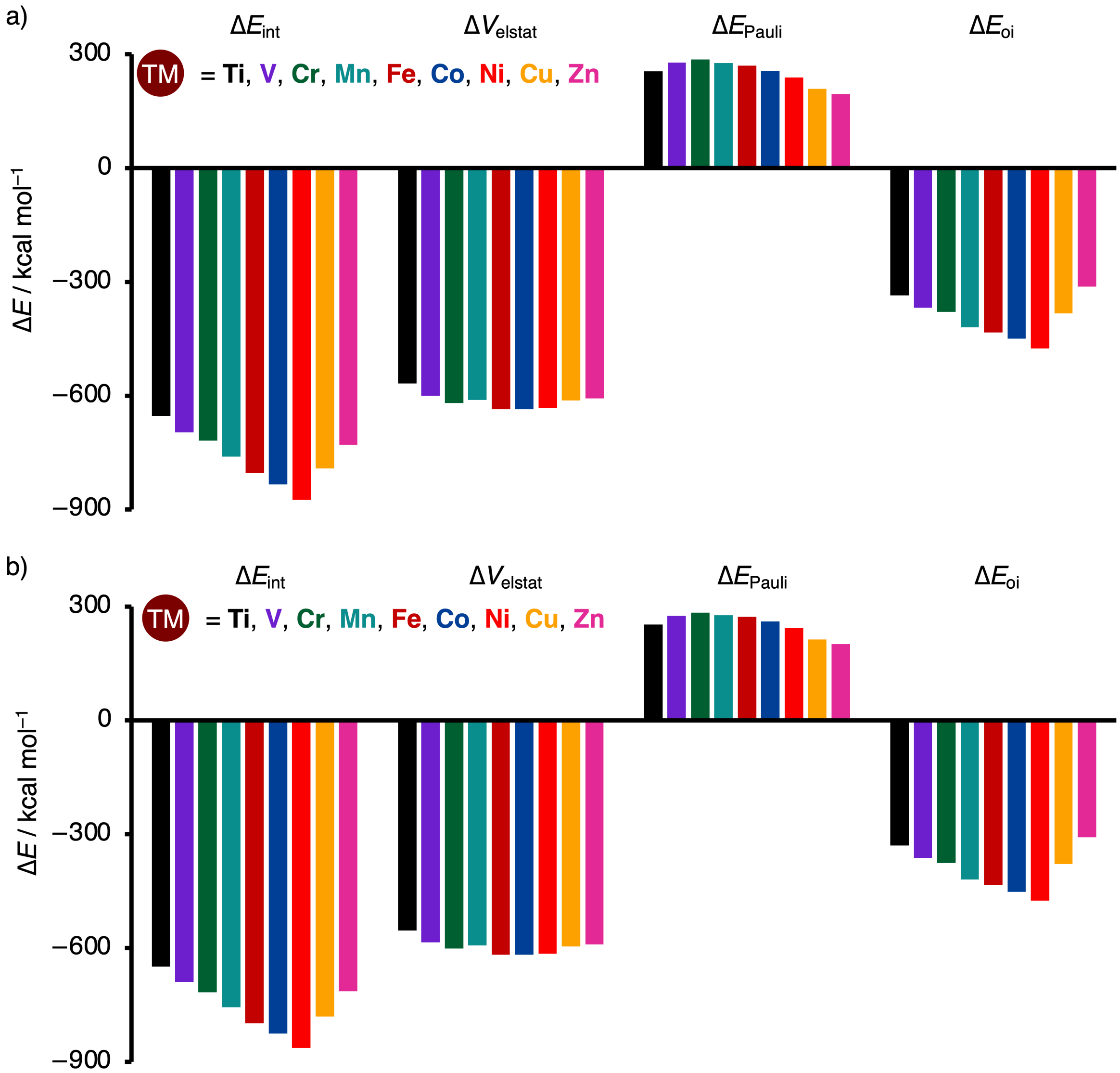

Figure S2. Energy decomposition analysis (in kcal mol^{-1}) for the interaction between the transition metal (**TM^{2+}**) and nitrogen-doped graphene support (**$4NDG^{2-}$**) in **TM-4NDG**, where TM = Ti, V, Cr, Mn, Fe, Co, Ni, Cu, and Zn, computed at a) ZORA-UPBE-D3(BJ)/TZP b) ZORA-UOPBE-D3(BJ)/TZP. See Tables S7 and S11 for numerical data.

Figure S3. Representation of fragment molecular orbitals of $\mathbf{TM^{2+}}$ and $\mathbf{4NDG^{2-}}$ (isovalue = 0.03 Bohr$^{-3/2}$), sorted by the irreducible representations of the C_{2v} point group and computed at ZORA-UPBE-D3(BJ)/TZP.

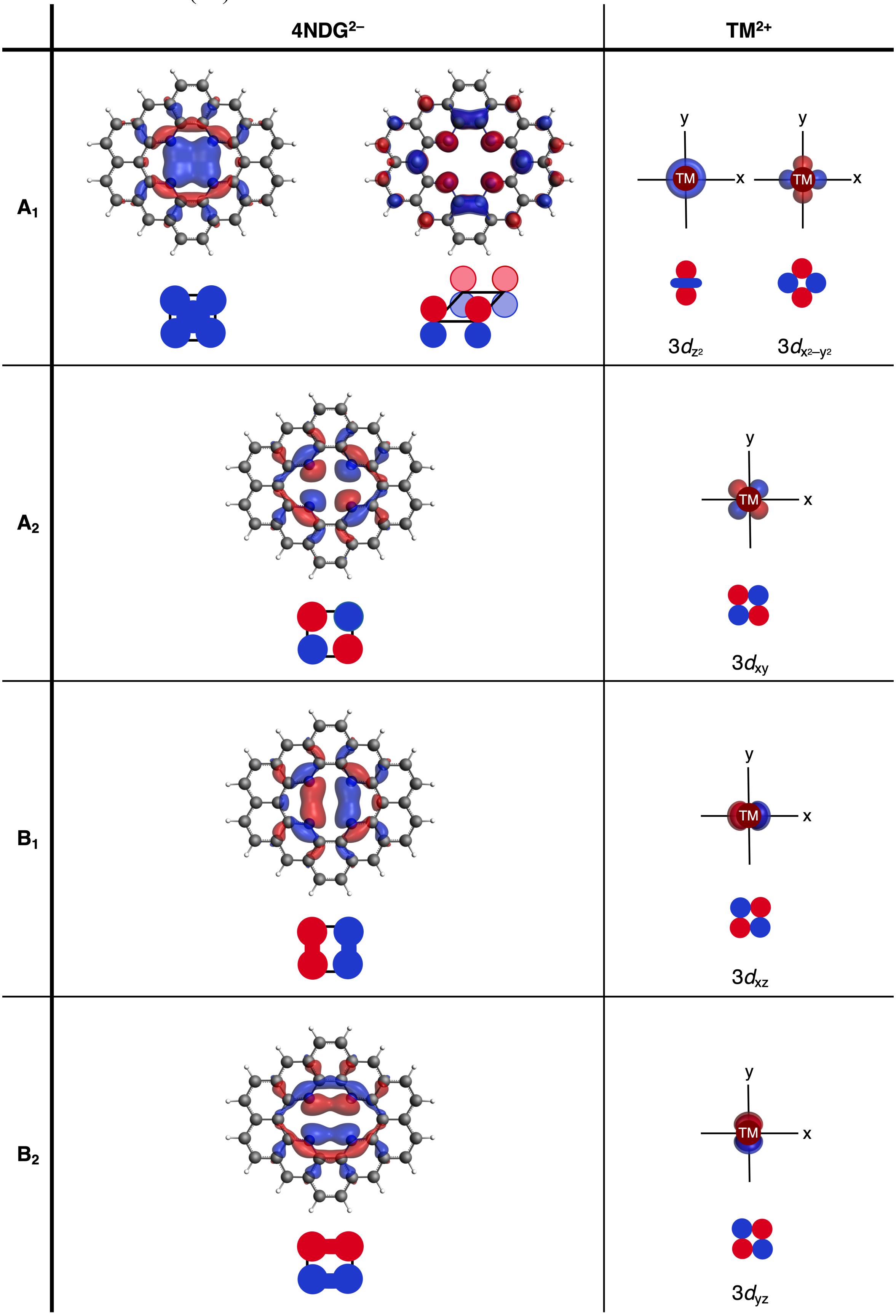

Table S5. Periodic activation strain analyses (in kcal mol^{-1}) for the interaction between the transition metal (**TM^{2+}**) and nitrogen-doped graphene support (**4NDG**) in **TM-4NDG**.[a]

TM-4NDG	ΔE	ΔE_{int}	ΔE_{strain}
**Ti-4NDG**	–385.4	–403.0	17.6
**V-4NDG**	–405.2	–417.3	12.2
**Cr-4NDG**	–445.6	–455.0	9.4
**Mn-4NDG**	–458.9	–470.1	11.2
**Fe-4NDG**	–493.2	–504.0	10.8
**Co-4NDG**	–521.3	–530.0	8.7
**Ni-4NDG**	–545.6	–554.5	8.9
**Cu-4NDG**	–507.5	–514.3	6.8
**Zn-4NDG**	–446.5	–454.9	8.4

[a] Computed with BAND at ZORA-UPBE-D3(BJ)/TZP.

Table S6. Activation strain analyses (in kcal mol^{-1}) for the interaction between the transition metal (**TM^{2+}**) and nitrogen-doped graphene support (**4NDG**) in **TM-4NDG**.[a]

TM-4NDG	ΔE	ΔE_{int}	ΔE_{strain}
**Ti-4NDG**	–640.7	–653.2	12.5
**V-4NDG**	–685.7	–696.4	10.7
**Cr-4NDG**	–710.0	–718.5	8.5
**Mn-4NDG**	–753.1	–760.4	7.3
**Fe-4NDG**	–793.4	–803.7	10.3
**Co-4NDG**	–823.1	–833.4	10.3
**Ni-4NDG**	–866.5	–875.2	8.7
**Cu-4NDG**	–785.7	–792.3	6.6
**Zn-4NDG**	–722.3	–730.1	7.7

[a] Computed at ZORA-UPBE-D3(BJ)/TZP.

Table S7. Energy decomposition analysis (in kcal mol^{-1}) for the interaction between the transition metal (**TM^{2+}**) and nitrogen-doped graphene support (**4NDG**) in **TM-4NDG**.[a]

TM-4NDG	ΔE_{int}	ΔV_{elstat}	ΔE_{Pauli}	ΔE_{oi}	ΔE_{disp}
**Ti-4NDG**	–653.2	–567.7	255.4	–335.3	–5.6
**V-4NDG**	–696.4	–601.0	277.9	–368.2	–5.1
**Cr-4NDG**	–718.5	–619.0	286.6	–379.7	–6.4
**Mn-4NDG**	–760.4	–611.0	276.6	–420.5	–5.5
**Fe-4NDG**	–803.7	–635.1	270.5	–433.4	–5.6
**Co-4NDG**	–833.4	–635.4	256.6	–449.7	–4.8
**Ni-4NDG**	–875.2	–633.0	238.4	–475.8	–4.8
**Cu-4NDG**	–792.3	–612.6	208.7	–383.0	–5.4
**Zn-4NDG**	–730.1	–607.4	195.2	–313.2	–4.7

[a] Computed at ZORA-UPBE-D3(BJ)/TZP.

Table S8. Orbital interaction energies decomposed into irreducible representation of C_{2v} (in kcal mol^{-1}) for the interaction between the transition metal (**TM^{2+}**) and nitrogen-doped graphene support (**4NDG**) in **TM-4NDG**.[a]

TM-4NDG	ΔE_{oi}	A_1	A_2	B_1	B_2
**Ti-4NDG**	–335.3	–84.7	–106.5	–70.1	–74.0
**V-4NDG**	–368.2	–99.1	–130.2	–80.4	–58.6
**Cr-4NDG**	–379.7	–103.2	–151.5	–61.2	–63.9
**Mn-4NDG**	–420.5	–112.8	–177.5	–71.6	–58.6
**Fe-4NDG**	–433.4	–112.1	–185.0	–77.2	–59.1
**Co-4NDG**	–449.7	–116.3	–205.6	–64.0	–63.9
**Ni-4NDG**	–475.8	–115.1	–230.4	–65.0	–65.4
**Cu-4NDG**	–383.0	–119.0	–130.6	–65.5	–67.9
**Zn-4NDG**	–313.2	–129.7	–47.4	–66.7	–69.4

[a] Computed at ZORA-UPBE-D3(BJ)/TZP.

Table S9. Change in Voronoi deformation density (VDD) atomic charge ΔQ (in electrons) due to the formation of the bond between the $\mathbf{TM^{2+}}$ and $\mathbf{4NDG^{2-}}$, decomposed into contributions stemming from Pauli repulsion and orbital interactions ($\Delta Q = \Delta Q_{\text{Pauli}} + \Delta Q_{\text{oi}}$) and into contributions from the various irreps (Γ) of the molecular point-group symmetry (C_{2v}).[a]

TM-4NDG	Fragment		A_1	A_2	B_1	B_2	Total
**Ti-4NDG**	$\mathbf{4NDG^{2-}}$	ΔQ	–0.059	0.289	0.129	0.142	0.501
	$\mathbf{Ti^{2+}}$		0.059	–0.289	–0.129	–0.142	–0.501
	$\mathbf{4NDG^{2-}}$	ΔQ_{Pauli}	–0.033	0.000	–0.017	–0.016	–0.066
	$\mathbf{Ti^{2+}}$		0.033	0.000	0.017	0.016	0.066
	$\mathbf{4NDG^{2-}}$	ΔQ_{oi}	–0.026	0.289	0.146	0.158	0.567
	$\mathbf{Ti^{2+}}$		0.026	–0.289	–0.146	–0.158	–0.567
**V-4NDG**	$\mathbf{4NDG^{2-}}$	ΔQ	0.073	0.386	0.186	–0.250	0.395
	$\mathbf{V^{2+}}$		–0.073	–0.386	–0.186	0.250	–0.395
	$\mathbf{4NDG^{2-}}$	ΔQ_{Pauli}	–0.036	0.000	–0.019	–0.007	–0.062
	$\mathbf{V^{2+}}$		0.036	0.000	0.019	0.007	0.062
	$\mathbf{4NDG^{2-}}$	ΔQ_{oi}	0.109	0.386	0.206	–0.243	0.457
	$\mathbf{V^{2+}}$		–0.109	–0.386	–0.206	0.243	–0.457
**Cr-4NDG**	$\mathbf{4NDG^{2-}}$	ΔQ	0.070	0.458	–0.028	–0.099	0.402
	$\mathbf{Cu^{2+}}$		–0.070	–0.458	0.028	0.099	–0.402
	$\mathbf{4NDG^{2-}}$	ΔQ_{Pauli}	–0.036	0.000	–0.015	–0.015	–0.067
	$\mathbf{Cu^{2+}}$		0.036	0.000	0.015	0.015	0.067
	$\mathbf{4NDG^{2-}}$	ΔQ_{oi}	0.106	0.458	–0.012	–0.084	0.468
	$\mathbf{Cu^{2+}}$		–0.106	–0.458	0.012	0.084	–0.468
**Mn-4NDG**	$\mathbf{4NDG^{2-}}$	ΔQ	0.140	0.574	0.061	–0.344	0.432
	$\mathbf{Mn^{2+}}$		–0.140	–0.574	–0.061	0.344	–0.431
	$\mathbf{4NDG^{2-}}$	ΔQ_{Pauli}	–0.036	0.000	–0.015	–0.006	–0.058
	$\mathbf{Mn^{2+}}$		0.036	0.000	0.015	0.006	0.058
	$\mathbf{4NDG^{2-}}$	ΔQ_{oi}	0.176	0.574	0.076	–0.337	0.489
	$\mathbf{Mn^{2+}}$		–0.176	–0.574	–0.076	0.338	–0.489
**Fe-4NDG**	$\mathbf{4NDG^{2-}}$	ΔQ	0.090	0.596	0.092	–0.251	0.528
	$\mathbf{Fe^{2+}}$		–0.090	–0.596	–0.092	0.251	–0.528
	$\mathbf{4NDG^{2-}}$	ΔQ_{Pauli}	–0.026	0.000	–0.015	–0.010	–0.051
	$\mathbf{Fe^{2+}}$		0.026	0.000	0.015	0.010	0.050

	**4NDG^{2-}**	ΔQ_{oi}	0.116	0.596	0.107	–0.241	0.578
	**Fe^{2+}**		–0.116	–0.596	–0.107	0.241	–0.578
**Co-4NDG**	**4NDG^{2-}**	ΔQ	0.126	0.674	–0.048	–0.154	0.597
	**Co^{2+}**		–0.126	–0.674	0.048	0.154	–0.597
	**4NDG^{2-}**	ΔQ_{Pauli}	–0.027	0.000	–0.010	–0.010	–0.046
	**Co^{2+}**		0.027	0.000	0.010	0.010	0.046
	**4NDG^{2-}**	ΔQ_{oi}	0.152	0.674	–0.038	–0.144	0.644
	**Co^{2+}**		–0.152	–0.674	0.038	0.145	–0.644
**Ni-4NDG**	**4NDG^{2-}**	ΔQ	0.113	0.781	–0.031	–0.108	0.754
	**Ni^{2+}**		–0.113	–0.781	0.032	0.108	–0.754
	**4NDG^{2-}**	ΔQ_{Pauli}	–0.013	0.000	–0.009	–0.009	–0.032
	**Ni^{2+}**		0.013	0.000	0.009	0.009	0.032
	**4NDG^{2-}**	ΔQ_{oi}	0.126	0.781	–0.022	–0.099	0.786
	**Ni^{2+}**		–0.126	–0.781	0.022	0.099	–0.786
**Cu-4NDG**	**4NDG^{2-}**	ΔQ	0.225	0.444	0.033	0.010	0.712
	**Cu^{2+}**		–0.224	–0.444	–0.033	–0.010	–0.712
	**4NDG^{2-}**	ΔQ_{Pauli}	–0.016	0.009	–0.008	–0.007	–0.022
	**Cu^{2+}**		0.016	–0.009	0.008	0.008	0.022
	**4NDG^{2-}**	ΔQ_{oi}	0.240	0.435	0.041	0.018	0.734
	**Cu^{2+}**		–0.240	–0.435	–0.041	–0.017	–0.734
**Zn-4NDG**	**4NDG^{2-}**	ΔQ	0.394	0.038	0.093	0.088	0.612
	**Zn^{2+}**		–0.394	–0.038	–0.093	–0.088	–0.612
	**4NDG^{2-}**	ΔQ_{Pauli}	–0.013	0.026	–0.007	–0.006	0.000
	**Zn^{2+}**		0.013	–0.026	0.007	0.007	0.000
	**4NDG^{2-}**	ΔQ_{oi}	0.406	0.012	0.100	0.094	0.612
	**Zn^{2+}**		–0.406	–0.012	–0.100	–0.094	–0.612

[a] Computed at ZORA-UPBE-D3(BJ)/TZP.

Symmetry-Decomposed Voronoi Deformation Density Analysis[1]

One of the applications of the VDD method is studying atomic charge changes (ΔQ) caused by the bonding between molecular fragments [Eq. (1)].

$$\Delta Q = -\int_{\text{Voronoi cell of A in molecule}} \left[\rho_{complex}(\boldsymbol{r}) - \sum_{fragmetns,i} \rho_{\text{i}}(\boldsymbol{r})\right] d\boldsymbol{r} \qquad (1)$$

Equation (1) describes the change in charge (ΔQ) in the final density of the overall complex, relative to the sum of the initial molecular fragment densities. This illustrates how the chemical bond between these subsystems alters the electron density distribution. Specifically, ΔQ indicates the amount of electronic charge that flows into ($\Delta Q < 0$) or out of ($\Delta Q > 0$) the Voronoi cell of an atom due to the interaction between the molecular fragments. A notable advantage of the VDD method is that by utilizing molecular symmetry ΔQ can be partitioned into contributions from the different irreducible representations (irreps) Γ of the point-group symmetry of the fragment-based promolecule [Eq. (2)] or a corresponding sub-point group. For C_{2V} symmetric molecular systems, this symmetry decomposition of the total ΔQ provides the insightful distinction between charge shifts within the A_1, A_2, B_1 and B_2 irreps.

$$\Delta Q = \sum_{\Gamma} \Delta Q^{\Gamma} \qquad (2)$$

The deformation density of the complex, and consequently the charge change ΔQ induced by the chemical bond, can be divided into contributions from Pauli repulsion (ΔQ_{Pauli}) and orbital interaction (ΔQ_{oi}). These contributions can further be partitioned according to the different irreps Γ of the point-group molecular symmetry [Eq. (3)]. This framework provides multiple analysis routes for decomposing ΔQ per atom into: (i) symmetry (irrep Γ) components; (ii) components from Pauli repulsion and orbital interaction; and (iii) a combination of both.

$$\Delta Q = \sum_{\Gamma} \Delta Q^{\Gamma}_{Pauli} + \Delta Q^{\Gamma}_{oi} \qquad (3)$$

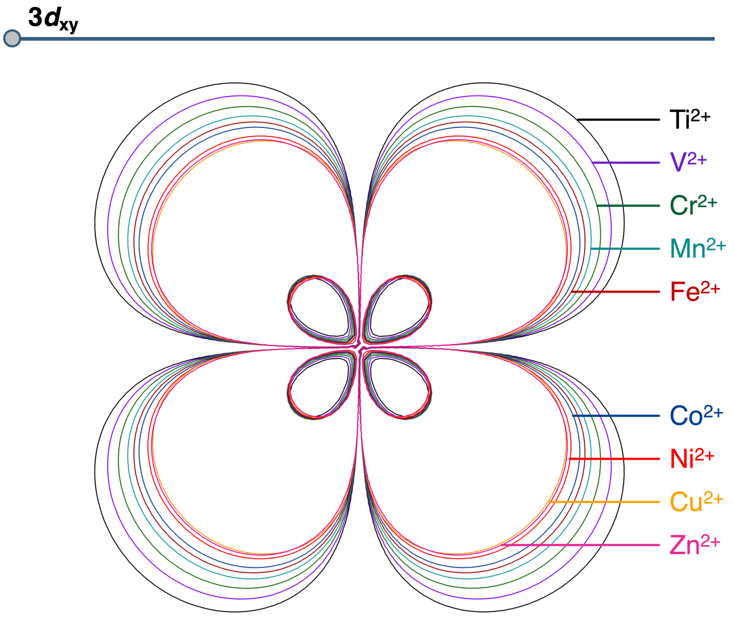

Figure S4. Contour plot of the atomic $3d_{xy}$ orbitals of the **TM^{2+}** fragment with **TM^{2+}= Ti^{2+}** (black), **V^{2+}** (purple), **Cr^{2+}** (green), **Mn^{2+}** (turquoise), **Fe^{2+}** (dark red), **Co^{2+}** (blue), **Ni^{2+}** (bright red), **Cu^{2+}** (orange), and **Zn^{2+}** (pink). All contour plots contain 2 contours from 0.0195 – 0.4000 au, computed at ZORA-UPBE-D3(BJ)/TZP.

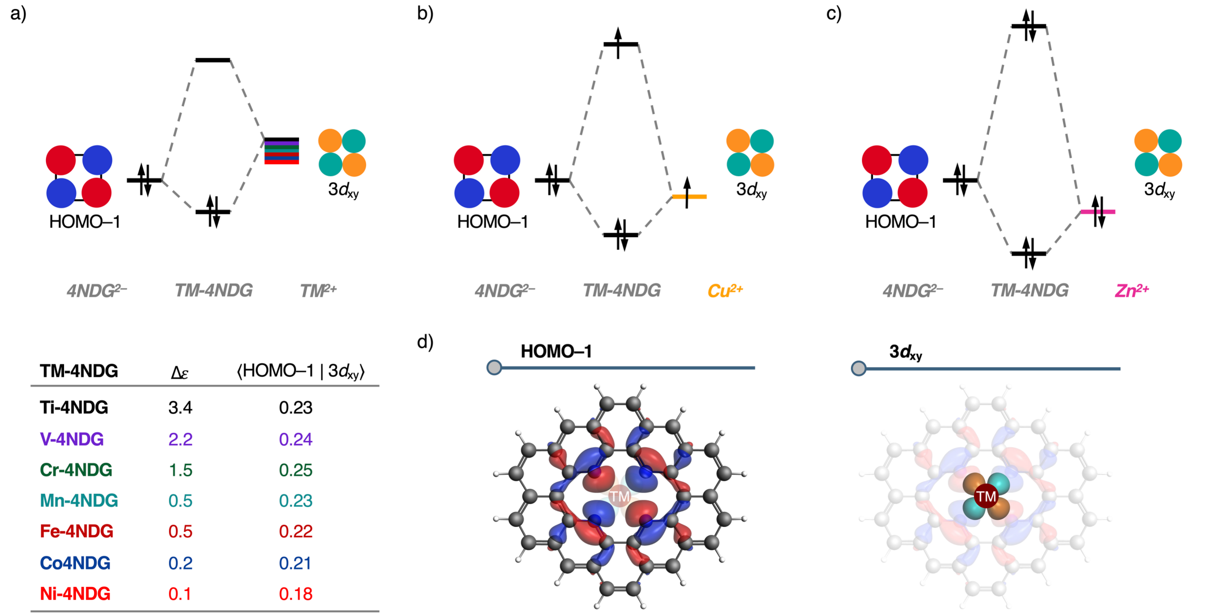

TM-4NDG	$\Delta\varepsilon$	⟨HOMO–1 | $3d_{xy}$⟩
Ti-4NDG	3.4	0.23
V-4NDG	2.2	0.24
Cr-4NDG	1.5	0.25
Mn-4NDG	0.5	0.23
Fe-4NDG	0.5	0.22
Co4NDG	0.2	0.21
Ni-4NDG	0.1	0.18

Figure S5. a) Schematic molecular orbital diagram and the overlaps and energy gaps (α-spin, in eV) of the HOMO–LUMO interaction between period 4 transition metal **TM^{2+}** and nitrogen-doped graphene **$4NDG^{2–}$**; Schematic molecular orbital diagram of b) the HOMO–SOMO interaction between **$4NDG^{2–}$** and **Cu^{2+}**; c) the HOMO–HOMO interaction between **$4NDG^{2–}$** and **Zn^{2+}** and d) key orbitals of **$4NDG^{2–}$** and **TM^{2+}** (isovalue = 0.03 $Bohr^{-3/2}$), computed at ZORA-UPBE-D3(BJ)/TZP.

Table S10. TM^{2+} and 4NDG^{2-} ground state occupations and valence occupations[a] expressed in irreducible representations of C_{2V}.

TM^{2+}	Ground State Occupations	Valence Occupations
**Ti^{2+}**	$A_1^{12}\ A_2^{0}\ B_1^{4}\ B_2^{4}$	$A_1^{2}\ A_2^{0}\ B_1^{0}\ B_2^{0}$
**V^{2+}**	$A_1^{12}\ A_2^{0}\ B_1^{4}\ B_2^{5}$	$A_1^{2}\ A_2^{0}\ B_1^{0}\ B_2^{1}$
**Cr^{2+}**	$A_1^{12}\ A_2^{0}\ B_1^{5}\ B_2^{5}$	$A_1^{2}\ A_2^{0}\ B_1^{1}\ B_2^{1}$
**Mn^{2+}**	$A_1^{12}\ A_2^{0}\ B_1^{5}\ B_2^{6}$	$A_1^{2}\ A_2^{0}\ B_1^{1}\ B_2^{2}$
**Fe^{2+}**	$A_1^{13}\ A_2^{0}\ B_1^{5}\ B_2^{6}$	$A_1^{3}\ A_2^{0}\ B_1^{1}\ B_2^{2}$
**Co^{2+}**	$A_1^{13}\ A_2^{0}\ B_1^{6}\ B_2^{6}$	$A_1^{3}\ A_2^{0}\ B_1^{2}\ B_2^{2}$
**Ni^{2+}**	$A_1^{14}\ A_2^{0}\ B_1^{6}\ B_2^{6}$	$A_1^{4}\ A_2^{0}\ B_1^{2}\ B_2^{2}$
**Cu^{2+}**	$A_1^{14}\ A_2^{1}\ B_1^{6}\ B_2^{6}$	$A_4\ A_2^{1}\ B_1^{2}\ B_2^{2}$
**Zn^{2+}**	$A_1^{14}\ A_2^{2}\ B_1^{6}\ B_2^{6}$	$A_1^{4}\ A_2^{2}\ B_1^{2}\ B_2^{2}$
**4NDG^{2-}**	$A_1^{74}\ A_2^{58}\ B_1^{66}\ B_2^{64}$	–

[a] Only the occupations of the 3d atomic orbitals are shown.

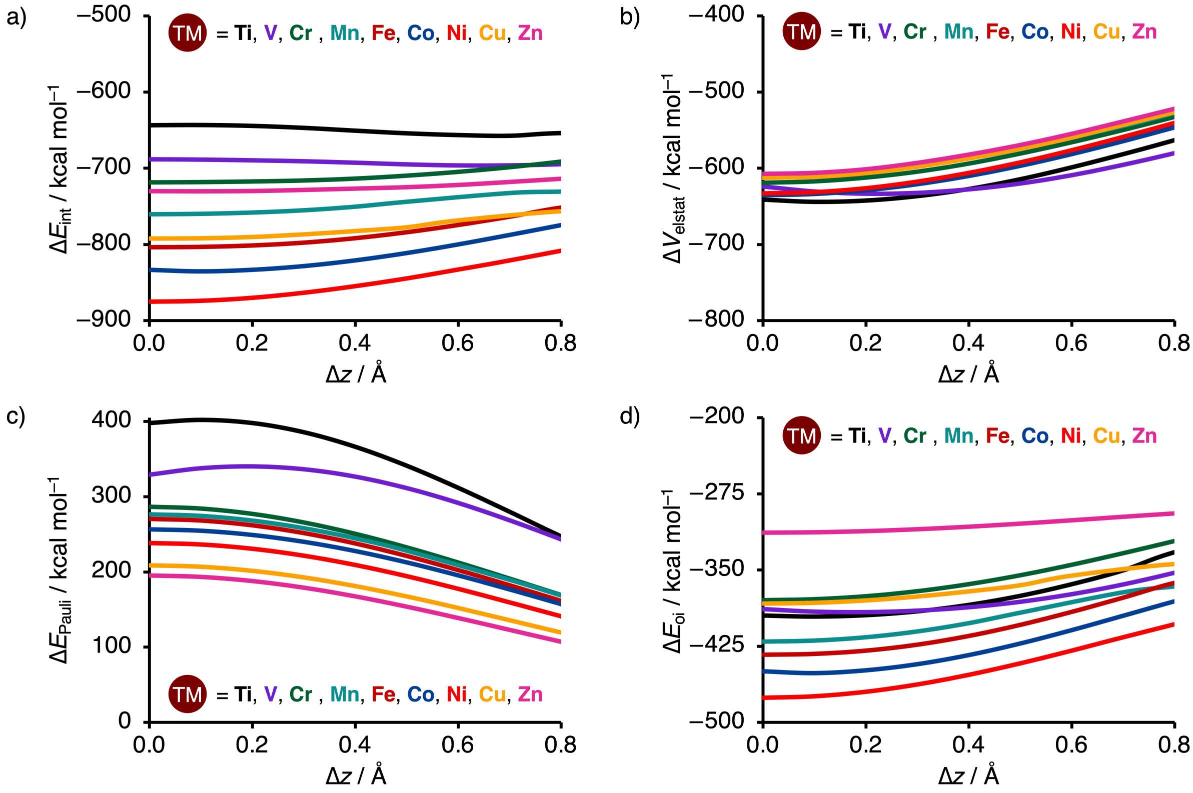

Figure S6. Energy decomposition analysis (in kcal mol^{-1}): a) interaction energy; b) electrostatic interaction; c) Pauli repulsion; and d) orbital interaction of the **TM^{2+}···4NDG^{2-}** bond (with TM = Ti, V, Cr, Mn, Fe, Co, Ni, Cu, and Zn) as function of the vertical displacement of **TM^{2+}** with respect to **4NDG^{2-}**, computed at ZORA-UPBE-D3(BJ)/TZP.

Table S11. Energy decomposition analysis (in kcal mol^{-1}) for the interaction between the transition metal (**TM^{2+}**) and nitrogen-doped graphene support (**4NDG**) in **TM-4NDG**, computed at ZORA-UOPBE-D3(BJ)/TZP.

TM-4NDG	ΔE_{int}	ΔV_{elstat}	ΔE_{Pauli}	ΔE_{oi}	ΔE_{disp}
**Ti-4NDG**	–648.7	–553.5	253.4	–330.3	–18.4
**V-4NDG**	–689.0	–584.4	275.3	–362.8	–17.1
**Cr-4NDG**	–716.1	–601.4	284.0	–375.6	–23.0
**Mn-4NDG**	–755.7	–593.1	276.7	–420.0	–19.3
**Fe-4NDG**	–798.6	–617.4	273.5	–434.5	–20.2
**Co-4NDG**	–825.9	–617.8	261.4	–452.1	–17.4
**Ni-4NDG**	–863.3	–615.2	243.6	–474.4	–17.3
**Cu-4NDG**	–780.1	–595.4	213.5	–378.6	–19.7
**Zn-4NDG**	–714.2	–590.2	200.9	–307.9	–17.1

Table S12. Orbital interaction energies decomposed into irreducible representations of C_{2V} (in kcal mol^{-1}) for the interaction between the transition metal (**TM^{2+}**) and nitrogen-doped graphene support (**4NDG**) in **TM-4NDG**, computed at ZORA-UOPBE-D3(BJ)/TZP.

TM-4NDG	ΔE_{oi}	A_1	A_2	B_1	B_2
**Ti-4NDG**	–330.3	–81.3	–105.4	–69.9	–73.7
**V-4NDG**	–362.8	–95.9	–129.5	–79.8	–57.7
**Cr-4NDG**	–375.6	–101.4	–151.1	–60.2	–63.0
**Mn-4NDG**	–420.0	–112.2	–180.2	–70.9	–56.8
**Fe-4NDG**	–434.5	–112.7	–187.2	–76.6	–58.1
**Co-4NDG**	–452.1	–116.6	–207.7	–63.8	–63.9
**Ni-4NDG**	–474.4	–112.9	–231.8	–64.6	–65.0
**Cu-4NDG**	–378.6	–115.1	–131.6	–64.7	–67.1
**Zn-4NDG**	–307.9	–125.3	–47.1	–66.4	–69.1

Table S13. Total energies of the **TM-4NDG** systems (in kcal mol^{-1}) optimized using C_1 and C_{2v} symmetry.[a]

TM-4NDG	C_1	C_{2v}	Difference in Energy
**Ti-4NDG**	–9785.7	–9780.0	5.7
**V-4NDG**	–9814.6	–9814.6	0.0
**Cr-4NDG**	–9854.2	–9854.1	0.1
**Mn-4NDG**	–9848.6	–9848.5	0.1
**Fe-4NDG**	–9834.4	–9834.2	0.2
**Co-4NDG**	–9818.3	–9816.4	1.9
**Ni-4NDG**	–9765.5	–9765.3	0.2
**Cu-4NDG**	–9695.7	–9695.6	0.1
**Zn-4NDG**	–9649.5	–9649.5	0.0

[a] Computed at ZORA-UPBE-D3(BJ)/TZP. For the C_1 energies the optimized geometries from BAND were used and only the C–H bonds were additionally optimized in ADF.

Table S14. Total energies of the **TM-4NDG** systems (in kcal mol^{-1}) optimized using periodic DFT (BAND) and DFT (ADF) with C_1 and C_{2v} symmetry.[a]

Systems	E_{BAND}	$E_{ADF}(C_{2v})$	$E_{ADF}(C_1)$
**4NDG^{2-}**	–14948.6	–9585.0	–9585.0
**Ti^{2+}**	327.8	449.3	449.3
**V^{2+}**	322.0	455.9	455.9
**Cr^{2+}**	326.3	440.6	440.6
**Mn^{2+}**	342.6	509.8	509.8
**Fe^{2+}**	392.1	543.8	543.8
**Co^{2+}**	435.5	591.3	591.3
**Ni^{2+}**	516.5	685.9	685.9
**Cu^{2+}**	544.7	675.0	675.0
**Zn^{2+}**	530.1	658.2	658.2
**Ti-4NDG**	–15006.2	–9773.6	–9785.7
**V-4NDG**	–15031.8	–9810.2	–9814.6
**Cr-4NDG**	–15067.8	–9854.1	–9854.2
**Mn-4NDG**	–15064.9	–9848.5	–9848.6
**Fe-4NDG**	–15049.7	–9834.2	–9834.4
**Co-4NDG**	–15032.4	–9816.4	–9818.3
**Ni-4NDG**	–14977.7	–9765.3	–9765.5
**Cu-4NDG**	–14911.4	–9695.6	–9695.7
**Zn-4NDG**	–14865.0	–9649.5	–9649.5

[a] Computed at ZORA-UPBE-D3(BJ)/TZP. For the C_1 energies the optimized geometries from BAND were used and only the C–H bonds were additionally optimized in ADF.

Table S15. Voronoi deformation density (VDD) atomic charge *Q* (in electrons) on the transition metal in **TM-4NDG** optimized using periodic DFT (BAND).[a]

TM-4NDG	VDD Charges
**Ti-4NDG**	0.411
**V-4NDG**	0.242
**Cr-4NDG**	0.150
**Mn-4NDG**	0.028
**Fe-4NDG**	0.259
**Co-4NDG**	0.399
**Ni-4NDG**	0.416
**Cu-4NDG**	0.411
**Zn-4NDG**	0.242

[a] Computed at ZORA-UPBE-D3(BJ)/TZP.

References

[1] a) C. Fonseca Guerra, J. W. Handgraaf, E. J. Baerends, F. M. Bickelhaupt*, J. Comput. Chem.* **2004**, *25*, 189; b) C. Nieuwland, P. Vermeeren, F. M. Bickelhaupt, C. Fonseca Guerra, *J. Comput. Chem.* **2023**, *44*, 2108.
